# Commentary: Association between metallic implants and stroke in US adults from NHANES 2015–2023 a cross-sectional study

**DOI:** 10.3389/fnagi.2025.1586693

**Published:** 2025-05-21

**Authors:** Xiangming Li, Peixing Huang, Guizhong Huang

**Affiliations:** Shantou Hospital of Traditional Chinese Medicine, Guangzhou University of Traditional Chinese Medicine, Shantou, Guangdong, China

**Keywords:** NHANES, question, metallic implants, stroke, commentary

With the development of modern medicine, metal-based biomedical implants are being increasingly and widely applied in clinical treatments. Examples include steel plates and screws in orthopedic surgeries, as well as stents in the treatment of cardiovascular diseases. However, implanted metallic devices may trigger a series of biological effects within the body, and their potential associations with certain diseases have gradually drawn attention. Stroke, a disease that seriously threatens human health, makes the exploration of the relationship between metal-containing prosthetic devices and stroke of great significance for evaluating the safety and potential risks of metallic implants. Against this backdrop, we have read with great interest the article titled “Association between metallic implants and stroke in US adults from NHANES 2015–2023: a cross - sectional study” recently published in Frontiers in Aging Neuroscience by Wu et al. (2024). This study utilized the data from the National Health and Nutrition Examination Survey (NHANES) in the United States from 2015 to 2023 to conduct a cross - sectional analysis of 12,337 American adults, providing crucial and profound insights into uncovering the complex relationship between metal-based implants and stroke. The study revealed that the stroke risk among individuals with internal metallic prostheses is higher compared to those without. Notably, women with metallic implants have a significantly higher stroke risk than men. This finding is both thought—provoking and clinically relevant. Although this study, by leveraging the NHANES database, has put forward valuable insights into the complex relationship between metallic medical devices and stroke, it is essential to recognize that there are some limitations that may affect the interpretation of the results.

## 1 Influence of racial distribution discrepancies on the generalizability of results

The study's reliance on NHANES data, while providing a comprehensive perspective on the health status of the US population, reveals significant disparities in the racial distribution between the group with metal-based prosthetic devices and the non-implant group. This may undermine the generalizability of the results. Although robust statistical methods were employed for data weighting and stratification, the inherent variability and potential biases within the larger population might not be fully captured. The article adopts a cross—sectional study design, which is a snapshot in time. While it can identify associations between implanted metallic devices and stroke, it cannot establish a causal relationship. It is challenging to determine whether presence of internal metal-containing implants precede stroke or vice versa, and the possibility that unmeasured factors simultaneously affect both cannot be excluded. This restricts a profound understanding of the nature of their relationship. Future research could consider using the Mendelian randomization approach to analyze the relationship between metal ion exposure due to implanted devices and stroke. Biomarkers associated with metal ions and stroke can be identified through bioinformatics and Mendelian randomization analyses.

## 2 Impact of key information deficiency on research results

The NHANES database lacks detailed records of metal-based biomedical implants. Crucial information such as the types of implants, their quantities, the duration of their presence in the body, as well as the corrosion rates and ion release characteristics of different metallic materials, is absent. This deficiency renders it impossible for the study to comprehensively evaluate the potential impact of different metal types on stroke risk. Implanted metal-containing devices vary in terms of their functions, biomechanical loads, and *in-vivo* degradation processes. These differences can influence the research results, enhance inter-group heterogeneity, and reduce the clinical specificity of the research conclusions.

## 3 Impact of unmeasured confounding factors on the reliability of results

Although the study incorporated some potential confounding factors, such as gender, age, race, poverty status, educational level, body mass index, smoking, diabetes, coronary heart disease, and hypertension, as covariates (Wu et al., 2024), there may still exist unmeasured or unknown confounding factors. These factors can lead to residual confounding effects in the study, interfering with the accurate assessment of the relationship between metallic implants and stroke and undermining the reliability of the research conclusions. For instance, potential confounding factors like cholesterol (Ma et al., 2025), triglycerides (Fu et al., 2025), cancer (Qu et al., 2023), sleep conditions (Huang et al., 2025), and dietary factors (Liu and Huang, 2025) could further enhance the reliability of the research results if they were further adjusted for.

## 4 Impact of incomplete subgroup analysis

The authors conducted a highly detailed subgroup analysis, yet failed to perform subgrouping based on race and socioeconomic status. Given that there are differences in stroke incidence or the prevalence of metal-based prosthetic devices among populations of different races and socioeconomic statuses (Cheng et al., 2024; Pantoja-Ruiz et al., 2025), this unbalanced distribution may still conceal specific associations within certain subgroups. As a result, the research conclusions may not accurately reflect the relationship between implanted metallic devices and stroke among populations of different races and socioeconomic statuses. We believe that adding subgroup analyses for different racial and socioeconomic status groups would be highly valuable.

## 5 Interference of blood heavy metal factors with research results

Implanted metallic devices in the body may release metal ions, thereby causing changes in the heavy metal content in the blood. For example, orthopedic implants such as cobalt-chromium alloys, when interacting with the human body's humoral environment over an extended period, release metal ions like cobalt and chromium into the bloodstream, increasing the concentration of relevant heavy metals in the blood (Houdek et al., 2024). This establishes a direct link between metal-containing prosthetic devices and elevated heavy metals in the blood. A study by Reiner et al. (2019) demonstrated that patients undergoing anatomic or reverse total shoulder arthroplasty (TSA) exhibited significantly higher blood metal ion concentrations compared to 23 healthy controls, with cobalt (0.18 μg/L vs. 0.11 μg/L), chromium (0.48 μg/L vs. 0.14 μg/L), and titanium (1.31 μg/L vs. 0.62 μg/L) levels showing statistically significant elevations postoperatively in a cohort of 40 TSA patients. A meta-analysis demonstrated significant increases in titanium (MD 0.81, 95% CI 0.32–1.30) and chromium levels (OR 23.50, 95% CI 5.56–99.31) following instrumented spinal surgery, with approximately 70% of patients showing elevated chromium levels (Burgos et al., 2024). Furthermore, emerging evidence indicates that fluctuations in blood concentrations of neurotoxic heavy metals (e.g., lead, cadmium) are significantly associated with stroke risk through multiple pathways (He et al., 2024). However, current evidence on systemic metal toxicity from surgical metal devices remains inconclusive. A large-scale review by Zywiel et al. (2016) noted that elevated blood metal levels from orthopedic implants rarely correlate with systemic cardiovascular complications in the general population. Similarly, studies focusing on hypersensitivity reactions to metal implants (Teo and Schalock, 2016) emphasize localized immune responses rather than systemic stroke risk. Since blood-borne heavy metals are both influenced by biomedical metal implants and implicated in stroke risk, failure to adequately account for them in research may confound the accurate assessment of the association between such implants and stroke. This could either exaggerate or obscure the true relationship, potentially leading to biased or misleading conclusions. While localized ion release from implanted metal components is plausible, its systemic clinical relevance to stroke pathogenesis appears limited based on current evidence. Future studies should prioritize direct biomarker measurements (serum metal ion levels) alongside rigorous adjustment for confounders to clarify whether observed associations reflect causation or residual confounding.

## 6 Impact of the limitations of stroke diagnosis methods

Furthermore, in this study, the diagnosis of stroke relied on self-reported measurements. The subjectivity and incompleteness of self-reporting have affected the research outcomes in multiple ways. On one hand, due to limitations in participants' memory or medical knowledge, misdiagnoses or case omissions are likely to occur. This affects the statistical count of stroke cases, interferes with the assessment of the strength of the association between surgical metal devices and stroke, and leads to biased conclusions. On the other hand, self-reporting tends to miss key information such as stroke types, onset times, and severity levels. This hinders the analysis of the relationship between implanted metal components and different types and stages of stroke, potentially concealing the true association between them. Additionally, inaccurate diagnoses can misclassify stroke cases. During subgroup analysis, this confuses the associations between metal-based medical devices and stroke in different subgroups, rendering the subgroup analysis results unreliable and unable to accurately present the risk differences among different populations.

In conclusion, while this study has offered valuable insights into the relationship between metallic implants and stroke, readers should take into account the aforementioned limitations when interpreting the results. Future research, if it can employ larger and more direct sample sizes along with detailed clinical data, will further validate the relevant conclusions and deepen our understanding of the intricate relationship between implanted metal components and stroke.

## References

[B1] Burgos J. Hevia E. Sanpera I. García V. Moreno M. T. de, S. Mariscal G.. (2024). Elevated blood metal ion levels in patients undergoing instrumented spinal surgery: a systematic review and meta-analysis. Spine J. 24, 947–960. 10.1016/j.spinee.2024.02.019 38437920

[B2] Cheng S. Zheng H. Wei Y. Lin X. Gu Y. Guo X.. (2024). Gut microbiome and stroke: A bidirectional mendelian randomisation study in east asian and european populations. Stroke Vasc. Neurol. 9, 623–630. 10.1136/svn-2023-002717 38296585 PMC11791640

[B3] Fu L. Xing Q. Wang X. Chen Y. Kong J. Li J.. (2025). Exploring the association between the TyG-WHtR index and the incidence of stroke in the obese population: Based on NHANES data from 1998 to 2018. J. Stroke Cerebrovasc. Dis. 34:108209. 10.1016/j.jstrokecerebrovasdis.2024.108209 39710082

[B4] He J. Zhang W. Zhao F. Wang M. Wang Z. Liang C.. (2024). Investigation of the relationship between lead exposure in heavy metals mixtures and the prevalence of stroke: a cross-sectional study. BMC Public Health 24:3474. 10.1186/s12889-024-21000-y 39696280 PMC11658309

[B5] Houdek M. T. Couch C. G. Wyles C. C. Taunton M. J. Rose P. S. Kremers H. M.. (2024). Whole blood metal levels in the setting of an oncologic endoprosthesis: is there cause for concern? Clin. Orthop. 482, 352–358. 10.1097/CORR.0000000000002805 37603308 PMC10776170

[B6] Huang J. Wu Y. Sun L. Liu Y. Wu S. Zhuang S.. (2025). Midday napping duration and risk of stroke: a prospective study in China. Sleep Med. 126, 205–210. 10.1016/j.sleep.2024.12.012 39700728

[B7] Liu J. Huang S. (2025). Dietary index for gut microbiota is associated with stroke among US adults. Food Funct. 16, 1458–1468. 10.1039/D4FO04649H 39898733

[B8] Ma H. X. Chen H. Q. Wang P. C. (2025). Association between non-high-density lipoprotein cholesterol to high-density lipoprotein cholesterol ratio (NHHR) and stroke among adults in the USA: a cross-sectional NHANES study. Biomed. Environ. Sci. 38, 37–46. 10.3967/bes2025.001 39924153

[B9] Pantoja-Ruiz C. Akinyemi R. Lucumi-Cuesta D. I. Youkee D. Emmett E. Soley-Bori M.. (2025). Socioeconomic status and stroke: a review of the latest evidence on inequalities and their drivers. Stroke 56, 794–805. 10.1161/STROKEAHA.124.049474 39697175 PMC11850189

[B10] Qu H. He C. Xu H. Sun X. (2023). Investigating the association of breast cancer and stroke: A two-sample mendelian randomization study. Medicine 102:e35037. 10.1097/MD.0000000000035037 37747009 PMC10519452

[B11] Reiner T. Bader N. Panzram B. Bülhoff M. Omlor G. Kretzer J. P.. (2019). In vivo blood metal ion levels in patients after total shoulder arthroplasty. J. Shoulder Elbow Surg. 28, 539–546. 10.1016/j.jse.2018.08.027 30518478

[B12] Teo W. Z. W. Schalock P. C. (2016). Metal hypersensitivity reactions to orthopedic implants. Dermatol. Ther. 7, 53–64. 10.1007/s13555-016-0162-1 27995484 PMC5336431

[B13] Wu K. Pang L. Su P. Lv C. (2024). Association between metallic implants and stroke in US adults from NHANES 2015–2023 a cross-sectional study. Front. Aging Neurosci. 16:1505645. 10.3389/fnagi.2024.1505645 39759400 PMC11695404

[B14] Zywiel M. G. Cherian J. J. Banerjee S. Cheung A. C. Wong F. Butany J.. (2016). Systemic cobalt toxicity from total hip arthroplasties: Review of a rare condition part 2. measurement, risk factors, and step-wise approach to treatment. Bone Jt. J. 98, 14–20. 10.1302/0301-620X.98B1.36712 26733510

